# Cryogenic Damage and Trehalose Protection in *Culter alburnus* Sperm: An Integrated Assessment of Quality, Physiology, and Protein Expression

**DOI:** 10.3390/ani16081245

**Published:** 2026-04-18

**Authors:** Shun Cheng, Shi-Li Liu, Mei-Li Chi, Wen-Ping Jiang, Jian-Bo Zheng, Chao Zhu, Jun-Zhi Luo, Fei Li

**Affiliations:** Key Laboratory of Healthy Freshwater Aquaculture, Zhejiang Institute of Freshwater Fisheries (Zhejiang Freshwater Fishery Environmental Monitoring Station), Ministry of Agriculture and Rural Affairs, Huzhou 313001, China; sschengshun@sina.com (S.C.); liushili1212@126.com (S.-L.L.);

**Keywords:** topmouth culter, sperm motility, enzymatic activities, sperm ultrastructure, proteomics

## Abstract

To address cryodamage in *Culter alburnus* sperm, this study evaluated the effects of trehalose supplementation in a conventional cryomedium (D-15 + 10% ethylene glycol). Six experimental groups were established: fresh sperm (G1), a conventional cryomedium (G2), groups supplemented with 10, 100, or 200 mmol/L trehalose (G3–G5), and a control group with extender only (G6). The group with 100 mmol/L trehalose (G4) was associated with improved post-thaw motility parameters (activation rate, movement time, and lifespan) and higher antioxidant (superoxide dismutase and catalase) and energy metabolism (ATPase, succinate dehydrogenase, lactate dehydrogenase) enzyme activities. Ultrastructural damage in G4 included partial plasma membrane rupture and mitochondrial swelling, while G6 exhibited additional damage features including membrane disintegration, mitochondrial disruption, and flagellar fracture. Proteomic analysis revealed that, compared to G1, G4 exhibited higher abundance of proteins (e.g., *Histone H2A*, *cytochrome c oxidase*, *profilin*) involved in structural integrity and energy homeostasis, whereas G6 showed signatures of oxidative stress and metabolic dysfunction (lower abundance of *NADH dehydrogenase* and higher abundance of *calcium-transporting ATPase* and *glutathione S-transferase*). In conclusion, 100 mmol/L trehalose was associated with improved cryopreservation outcomes, and the proteins identified provide a basis for further investigation. This approach offers a framework for refining germplasm conservation strategies in aquaculture.

## Simple Summary

This study addressed the high sensitivity of *Culter alburnus* sperm to cryopreservation-induced damage, aiming to optimize cryoprotection by integrating trehalose into the freezing medium. We systematically evaluated the effects of different trehalose concentrations (0–200 mmol/L) on post-thaw sperm quality. Results demonstrated that 100 mmol/L trehalose was associated with improved sperm motility (activation rate, movement time, and lifespan), better preservation of antioxidant and energy metabolism enzyme activities, and reduced structural damage to the plasma membrane, mitochondria, and flagella. Proteomic analysis revealed higher abundance of proteins associated with chromatin stability, mitochondrial function, and cytoskeletal integrity in this group, contrasting with markers of oxidative stress and metabolic disruption in unprotected sperm. We note that the individual contributions of trehalose and ethylene glycol cannot be separated in this study design, and fertilization trials are needed to confirm reproductive competence. Nevertheless, the findings identify a promising formulation for further evaluation and provide candidate protein markers for assessing cryopreservation success. This integrated approach offers a framework for refining germplasm conservation strategies for this species.

## 1. Introduction

*Culter alburnus* is an economically valuable freshwater fish in China; its industrialization development potential is enormous, and the research on the preservation of germplasm resources of this variety is of great significance [1,2]. Sperm cryopreservation is a pivotal technique for safeguarding genetic diversity and providing high-quality breeding material for genetic improvement programs [3]. There have been many studies on sperm cryopreservation in Cyprinidae fishes. Chen et al. [3] used D-15 as a diluent, 8–12% dimethyl sulfoxide as an antifreeze, and cryopreserved the sperm of *Ctenopharyngodon idellus*, *Hypophthalmichthys molitrix*, *Cyprinus carpio*, and *Megalobrama amblycephala*. Sperm activation rates reduced to 75%, 75%, 65%, and 65%, respectively. However, research focused on species within the Culterinae subfamily, including *C. alburnus*, remains limited. Preliminary work by our team identified a cryomedium comprising D-15 diluent and 10% ethylene glycol (EG) as a promising baseline for *C. alburnus* sperm [4,5].

The freeze–thaw process inevitably exposes spermatozoa to severe physicochemical stresses, leading to structural and functional impairments collectively termed cryodamage [6]. This damage is primarily attributed to the loss of plasma membrane integrity, induction of oxidative stress via reactive oxygen species (ROS), and disruptions to key metabolic and epigenetic pathways, ultimately compromising post-thaw viability and motility [7].

Trehalose is a typical stress metabolite that can form a unique protective film on the surface of cells under harsh environmental conditions, such as extreme temperature, altitude, osmotic pressure, and dehydration, effectively protecting the structure of biomolecules from damage and maintaining the life processes and biological characteristics of living organisms [8]. Exogenous trehalose also has nonspecific protective effects on organisms. Its protective mechanism is generally believed to be the strong binding of water molecules by the parts of the body containing trehalose, which, together with membrane lipids, possess bound water or trehalose itself, acting as a substitute for membrane-bound water, thereby preventing denaturation of biological membranes and membrane proteins [9]. Therefore, trehalose has been applied as a cryoprotective additive in the cryopreservation of *Epinephelus moara* and *Acipenser schrenckii* sperm [10,11]. Despite these promising applications, the specific molecular mechanisms by which trehalose confers protection in *C. alburnus* sperm remain poorly understood.

A comprehensive assessment of sperm quality post-thaw requires a multi-parametric approach that extends beyond conventional motility analysis (e.g., sperm activation rate, movement time, and lifespan). Biochemical and molecular evaluations provide deeper insights into cellular homeostasis. Enzymatic assays, for instance, offer a rapid and sensitive means to gauge cellular stress [12]. Key indicators include antioxidant enzymes like superoxide dismutase (SOD) and catalase (CAT), which counteract ROS [13,14,15], and energy metabolism markers such as ATPase, succinate dehydrogenase (SDH), and lactate dehydrogenase (LDH), which reflect mitochondrial integrity and ATP-generating capacity [16,17]. Studies on *A. schrenckii* sperm have confirmed significant reductions in these enzyme activities following cryopreservation, correlating with functional decline [11].

Ultrastructural analysis via scanning and transmission electron microscopy (SEM/TEM) visually delineates the physical sites of cryodamage. Consistent findings across species, including *C. carpio* and *Pseudosciaena crocea*, highlight vulnerabilities in the plasma membrane, mitochondrial architecture, and the flagellar axoneme, damage that directly impairs motility, energy production, and structural integrity [18,19,20,21]. Such observations confirm that membrane system and organelle dysfunction are central to cryoinjury. Chen et al. [11] observed and compared the morphology of *A. schrenckii* sperm before and after cryopreservation and found that the freezing damage to its structure was mainly concentrated in (1) membrane system damage, including acrosome membrane, cytoplasmic membrane, nuclear membrane, mitochondrial outer membrane and cristae membrane, and flagellar outer membrane; and (2) organelles, including mitochondrial enlargement and deformation and axonal shedding. Studies have also shown that after cryopreservation, most of the sperm of *Sebastes schgelii* exhibited structural damage such as mitochondrial cristae and cell membrane damage, as well as tail flagellar rupture [22]. After cryopreservation and thawing, the cytoplasmic and mitochondrial membranes of *Silurus lanzhouensis* sperm were damaged and vesicular, and the mitochondrial structure was dispersed, with neutrophil complex translocation [23]. Zhang et al. [24] found that membrane damage was the main cause of sperm freezing damage in *Scophthalmus maximus*, with 60–70% of damaged sperm exhibiting membrane damage. The structural damage of mitochondrial cristae deformation and plasma membrane expansion in frozen sperm of *Andrias davidianus* directly led to a decrease in the recovery rate and vitality of frozen sperm [25]. Therefore, it could be seen that although the types of fish were different, the freezing damage of fish sperm caused by cryopreservation was mainly manifested as damage to the membrane system and organelles.

Proteomics offers a powerful, systems level perspective by identifying global protein expression changes. Cryopreservation-induced alterations in the sperm proteome can reveal critical pathways involved in stress response, energy metabolism, cytoskeletal dynamics, and apoptosis [26]. For example, proteomic studies on carp sperm have linked motility to proteins involved in ubiquitination, glycolysis, and oxidative stress response, providing a molecular framework for understanding post-thaw function [27]. The study of Mariola et al. [6] showed that the majority of released proteins were involved in metabolism and energy production. Moreover, proteins associated with a response to stress, apoptosis, small GTPase, mediated signal transduction, transcription, translation, protein folding and turnover, reproduction, and DNA repair were identified. Therefore, applying this technology to *C. alburnus* can uncover key protein biomarkers of cryodamage and protection, informing mechanism driven optimization of cryoprotectants.

Given the limited research on *C. alburnus* sperm cryobiology and the need to elucidate the protective effects associated with trehalose, this study was designed to integrate functional, biochemical, structural, and proteomic analyses. We hypothesize that trehalose supplementation at an optimal concentration, would be associated with reduced cryodamage in *C. alburnus* sperm, potentially by preserving antioxidant capacity, maintaining mitochondrial energy metabolism, and stabilizing cellular ultrastructure, which is reflected in a distinct, protective proteomic profile. To test this, we evaluated the effects of different trehalose concentrations (0, 10, 100, 200 mmol/L) added to a base cryomedium (D-15 + 10% ethylene glycol (EG)) on post-thaw sperm motility, enzymatic activities, ultrastructure, and proteomic profile. The findings aim to identify a candidate cryoprotective formulation and provide molecular insights that may inform germplasm preservation strategies for this valuable species.

## 2. Materials and Methods

### 2.1. Materials

Experiments were conducted at the Zhejiang Institute of Freshwater Fisheries, Huzhou, China. *C. alburnus* used in this study were bred by Zhejiang Institute of Freshwater Fisheries, Huzhou, China. *C. alburnus* with good selective maturity were induced to labor by injecting luteinizing hormone-releasing hormone analog (LRH-A_2_) and chorionic gonadotropin (HCG) into the base of the pectoral fin. Sperm was collected by abdominal extrusion approximately 10 h later. This study was approved by the Ethics Committee of Laboratory Animal Center of Zhejiang Institute of Freshwater Fisheries (ZIFF20240405). The ethical approval date for this experiment was 5 April 2024.

### 2.2. Methods

#### 2.2.1. Sperm Collection

Prior to experimentation, fish were acclimated in a recirculating aquaculture system at 22 ± 1 °C. For semen collection, fish were anesthetized, and the abdominal surface was dried thoroughly. Milt was collected via gentle abdominal massage into sterile centrifuge tubes, which were immediately placed on ice (4 °C) and protected from light. Only samples exhibiting a milky-white, viscous consistency and an initial motility > 85% under microscopic examination were used for subsequent experiments.

#### 2.2.2. Preparation of Cryomedium

The basic extender (D-15) was prepared according to previous studies [4,5] with slight modifications: 0.8 g NaCl, 0.05 g KCl, and 1.5 g glucose were dissolved in 100 mL of ultrapure water (pH 7.2) and stored at 4 °C.

A total of six experimental groups were established:

Group 1 (control group, G1): untreated fresh sperm;

Group 2 (basic cryopreservation group, G2): D-15 extender + 10% (*v*/*v*) EG;

Group 3 (10 mmol/L trehalose treatment group, G3): G2 formulation supplemented with 10 mmol/L trehalose;

Group 4 (100 mmol/L trehalose treatment group, G4): G2 formulation supplemented with 100 mmol/L trehalose;

Group 5 (200 mmol/L trehalose treatment group, G5): G2 formulation supplemented with 200 mmol/L trehalose;

Group 6: (positive control group, G6): D-15 extender alone, without any cryoprotectant.

#### 2.2.3. Freezing and Thawing Protocol

Semen was diluted with the respective cryomedium at a 1:5 (*v*/*v*) ratio on ice. After a 10 min equilibration period at 4 °C, the mixture was aliquoted into 0.25 mL straws (0.2 mL each). The straws were placed on a rack 4 cm above the surface of liquid nitrogen (LN_2_) in a styrofoam box (vapor phase, approx. −80 to −100 °C) for 5 min before being plunged directly into LN_2_ (−196 °C) for long-term storage, following the procedure described by Cheng et al. [20]. For thawing, straws were rapidly retrieved from LN_2_ and immersed in a 40 °C water bath with gentle agitation until the ice crystal completely disappeared (approximately 10 s).

#### 2.2.4. Assessment of Sperm Motility Parameters

Sperm motility was initiated using deionized water. A 10 µL aliquot of thawed (or fresh) semen was mixed with 100 µL of deionized water on a glass slide and immediately examined under a phase-contrast microscope (Olympus BX53, Olympus Corporation, Tokyo, Japan) coupled with a digital camera for recording.

Activation rate (%): the percentage of motile sperm in a single field of view at 15 s post-activation.

Movement time (s): the time from activation until 90% of sperm exhibited only local vibration.

Lifespan (s): the time from activation until 90% of sperm ceased all movement.

Sperm motility was assessed subjectively under phase-contrast microscopy (Olympus BX53M, Olympus Corporation, Tokyo, Japan, 200×) by a single experienced observer blinded to treatment groups. Activation rate was defined as the percentage of sperm exhibiting any visible flagellar movement within a microscopic field, averaged across at least three fields per sample. Each measurement was performed with six independent biological replicates (*n* = 6). While we acknowledge that subjective assessment has limitations, the use of blinded analysis and multiple biological replicates per group provides consistent and reproducible comparative data.

#### 2.2.5. Biochemical Assays

All post-thaw analyses were performed immediately upon thawing. Motility was assessed within 10 s of activation; samples for enzyme activity and proteomic analysis were processed without delay to minimize time-dependent changes. Semen samples were centrifuged at 1000× *g* for 15 min at 4 °C. The seminal plasma was discarded, and the sperm pellet was gently resuspended and washed twice with ice-cold physiological saline (0.9% NaCl). After the final wash, the pellet was resuspended in saline to the original volume. Cell lysis was achieved by three freeze–thaw cycles (−20 °C/room temperature). The lysate was centrifuged at 12,000× *g* for 15 min at 4 °C, and the supernatant was collected for analysis.

The activities of key enzymes and metabolites were determined using commercial assay kits following the manufacturer’s protocols. Absorbance was measured with a spectrophotometer (Mapada UV-1200, Mapada Instruments, Shanghai, China).

Antioxidant status: SOD and CAT activities.

Energy metabolism: ATPase, SDH, and LDH activities.

SOD, CAT, ATPase, SDH, and LDH kits all purchased from Nanjing Jiancheng Bioengineering Institute, Nanjing, China (Catalog Nos. A001-1, A007-1, A016-1, A022-1, A020-2, respectively). All enzyme activities were normalized to total protein content measured by BCA assay (Catalog No. A045-4, same manufacturer).

All assays were conducted with six biological replicates (*n* = 6).

We acknowledge that structural damage in frozen-thawed sperm may lead to progressive enzyme leakage during centrifugation, potentially influencing measured activities. To minimize this, all samples were maintained at 4 °C throughout processing with identical centrifugation protocols, allowing valid relative comparisons between groups.

#### 2.2.6. Ultrastructural Observation by Electron Microscopy

Scanning electron microscopy (SEM): semen was fixed in 2.5% glutaraldehyde in 0.1 M PBS (pH 7.4) overnight at 4 °C. After PBS rinses, samples were dehydrated through a graded ethanol series (30–100%), subjected to tert-butanol substitution, and critical-point dried. Samples were sputter-coated with gold before observation under SEM.

Transmission electron microscopy (TEM): glutaraldehyde-fixed samples were post-fixed in 1% osmium tetroxide for 2 h at 4 °C, washed four times with PBS, dehydrated with 50%, 70%, 80%, and 90% ethanol for 15 min each, dehydrated twice with 100% ethanol for 20 min each time, and finally embedded in epoxy resin. After resin polymerization (hardening through heat curing), the blocks were trimmed, sectioned, stained, and examined under TEM.

A minimum of six independent samples per group were examined. For SEM, a minimum of 30 sperm cells from 3 biological replicates (10 sperm per replicate) were examined per group to confirm representativeness. For TEM, approximately 20–30 flagellar cross-sections, 15–20 mitochondria, and 10–15 nuclei were examined per group across 3 biological replicates.

#### 2.2.7. Proteomic Analysis

Sample preparation for proteomics: To investigate the core mechanisms of cryodamage and protection, proteomic analysis was performed on post-thaw, non-activated sperm from three critical groups: G1 (fresh sperm), G4 (optimal: 100 mmol/L trehalose), and G6 (maximum damage sperm). Each group consisted of six biological replicates (*n* = 6), each derived from a distinct male individual. Protein extraction and quantification refer to the method of Yang et al. [28]. Sperm pellets were washed with PBS and lysed in SDT buffer. Lysis was assisted by sonication on ice (80 W, 10 cycles of 10 s on/10 s off). After boiling and centrifugation, the protein concentration in the supernatant was determined using the BCA method.

4D label-free quantitative proteomics: Equal amounts of protein from each sample were digested using the FASP method with trypsin [29]. The resulting peptides were desalted, lyophilized, and reconstituted in 0.1% formic acid. LC-MS/MS analysis was performed on a timsTOF Pro 2 mass spectrometer (Brooke (Beijing) Technology Co., Ltd, Beijing, China) coupled to a nanoElute UPLC system. Peptides were separated on a reversed-phase C18 column with a linear acetonitrile gradient. Data were acquired in data-dependent acquisition (DDA) mode. Raw data files were processed using MaxQuant software (version 2.4.2). MS/MS spectra were searched against the *C. alburnus* protein database (UniProtKB/Swiss-Prot, release 2023_03, containing 52,428 entries). Cross-species annotation was justified by the high sequence conservation of core cellular proteins between cyprinids, with an average sequence identity of >85% for proteins identified in this study. The search parameters included: precursor mass tolerance of 10 ppm, fragment mass tolerance of 0.05 Da, up to two missed cleavages, carbamidomethylation of cysteine as a fixed modification, and methionine oxidation as a variable modification. Each of the six biological replicates per group was analyzed individually. For statistical analysis, differentially expressed proteins (DEPs) were identified with stringent thresholds: |Fold Change| > 2.0 and a *p*-value < 0.05 (adjusted using the Benjamini–Hochberg method) were considered significantly differentially abundant.

Bioinformatics analysis: DEPs were subjected to the following processes:

Gene ontology (GO) annotation: functional classification (biological process (BP), cellular component (CC), molecular function (MF)) was performed using Blast2GO (Version 5.2.5).

KEGG pathway enrichment analysis: pathway enrichment was conducted using the KOBAS-i (Version 3.0) tool. Terms with a *p*-value < 0.05 were considered significantly enriched.

Protein–protein interaction (PPI) network analysis: DEPs were submitted to the STRING database (Version 11.5, confidence score > 0.7). The resulting network was visualized and analyzed using Cytoscape (Version 3.9.1) software to identify key hub proteins and functional modules.

### 2.3. Statistical Analysis

Movement time was recorded as duration from activation until 90% of sperm ceased progressive movement. All data are presented as mean ± standard error (SEM). After arcsine transformation, the data obtained for each experiment were analyzed by analysis of variance using SPSS software (version 17.0; IBM, Armonk, NY, USA) to determine differences between groups. Tukey’s multiple comparison post hoc test was performed when significance was detected. Statistical significance was set at *p* ≤ 0.05.

## 3. Results

### 3.1. Post-Thaw Sperm Motility Parameters

**Figure 1:**
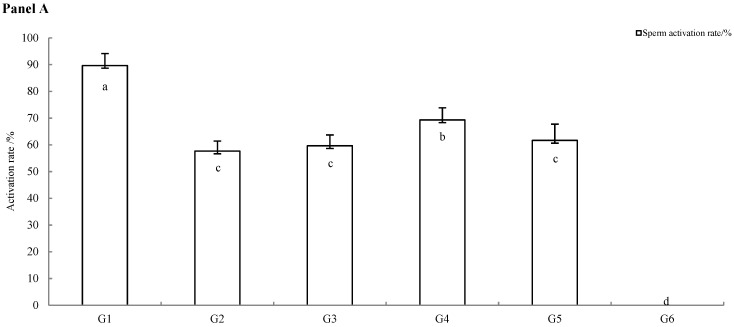

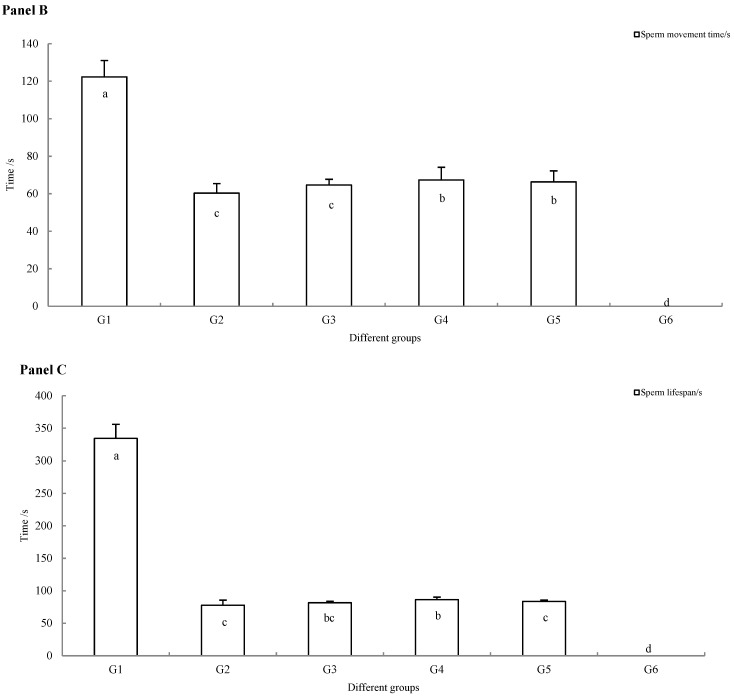
Effect of *C. alburnus* sperm activation rate (Panel (**A**)), movement time (Panel (**B**)), and lifespan (Panel (**C**)) of different groups. The x-axes are labeled with numerical identifiers (G1–G6) indicating the corresponding treatment groups (Groups 1–6). The data is average ± standard (*n* = 6); the rows marked with different letters are significantly different (*p* < 0.05).

The results showed that the effects of different cryoprotectant formulations on post-thaw sperm quality are summarized in Figure 1. As expected, sperm activation rate, movement time, and lifespan in the control group (G1) were significantly higher than in all cryopreserved groups (*p* < 0.05). Among the cryopreserved groups, the formulation supplemented with 100 mmol/L trehalose (G4) yielded favorable outcomes. The sperm activation rate in G4 was significantly higher than in groups G2, G3, G5, and G6 (*p* < 0.05). Furthermore, sperm from G4 exhibited a significantly longer movement time than those from G2 and G6 (*p* < 0.05), and a significantly extended lifespan compared to G2, G5, and G6 (*p* < 0.05).

In contrast, sperm cryopreserved in the extender alone without any cryoprotectant (G6) showed the most severe impairment, with all three motility parameters being significantly lower than in all other groups (*p* < 0.05).

### 3.2. Sperm Enzyme Activities

**Figure 2:**
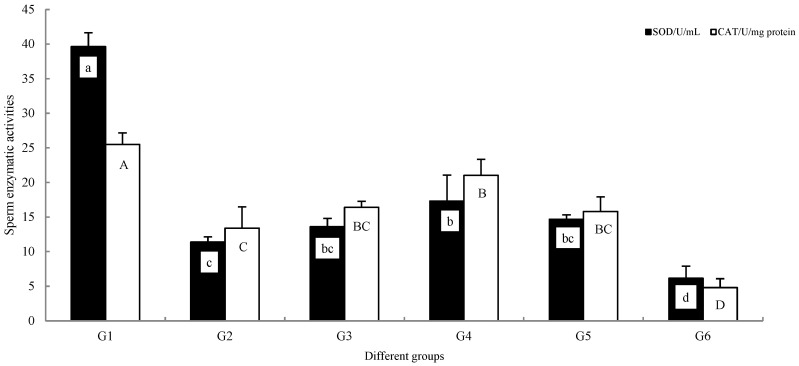
SOD and CAT detection of different groups of *C. alburnus* sperm. The x-axes are labeled with numerical identifiers (G1–G6) indicating the corresponding treatment groups (Groups 1–6). The data is average ± standard (*n* = 6); the rows marked with different letters are significantly different (*p* < 0.05).

**Figure 3:**
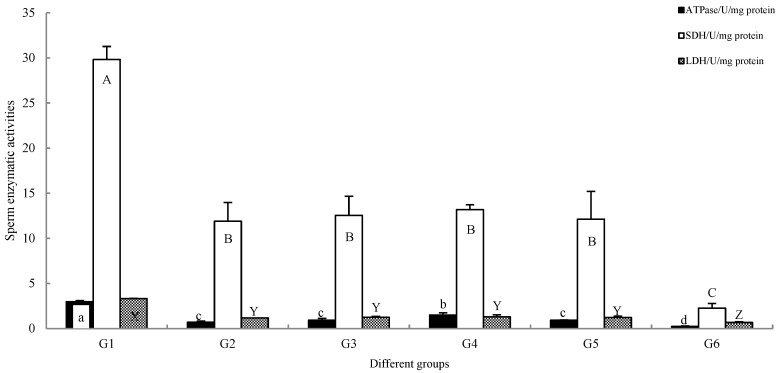
ATPase, SDH, and LDH detection of different groups of *C. alburnus* sperm. The x-axes are labeled with numerical identifiers (G1–G6) indicating the corresponding treatment groups (Groups 1–6). The data is average ± standard (*n* = 6); the rows marked with different letters are significantly different (*p* < 0.05).

Enzyme activity assays revealed significant differences among groups in both antioxidant capacity and energy metabolism (Figure 2 and Figure 3).

Antioxidant status: activities of SOD and CAT were highest in the G1, significantly exceeding all cryopreserved groups (*p* < 0.05). Among the frozen-thawed groups, G4 maintained significantly higher SOD and CAT levels compared to the G2 and G6 groups (*p* < 0.05). G6 showed significantly lower values than all other groups (*p* < 0.05).

Energy metabolism: a similar pattern was observed for key energy metabolism indicators, including ATPase, SDH, and LDH activities. All three parameters were significantly highest in G1 (*p* < 0.05). ATPase activity in G4 was significantly higher than in groups G2, G3, G5, and G6 (*p* < 0.05). While the SDH and LDH activities in G4 were not statistically different from G2, G3, and G5 (*p* > 0.05), they were numerically higher and were significantly greater than those in G6 (*p* < 0.05). G6 exhibited the lowest values for all three energy metabolism markers, significantly below all other groups (*p* < 0.05).

Unless otherwise noted, reported differences are statistically significant at *p* < 0.05. Non-significant trends are described using neutral language without attribution of biological meaning.

### 3.3. Ultrastructural Observation

Based on the results of sperm viability and enzyme activity, G4 was selected, alongside Group 1 (G1, fresh control) and Group 6 (G6, damage control), for detailed ultrastructural and proteomic analysis to elucidate the mechanisms of cryoprotection and cryodamage.

#### 3.3.1. SEM Observation of Sperm Ultrastructure

**Figure 4:**
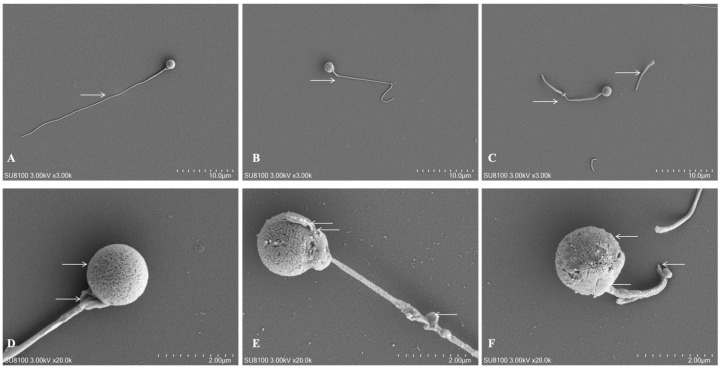
SEM observation of *C. alburnus* sperm ultrastructure. (**A**) SEM of complete morphology of Group 1 sperm (→ normal flagellum). (**B**) SEM of complete morphology of Group 4 sperm (→ curly flagellum). (**C**) SEM of complete morphology of Group 6 sperm (→ damaged and detached flagellum). (**D**) SEM of head and midpiece morphology of Group 1 sperm (→ normal head and midpiece). (**E**) SEM of head and midpiece morphology of Group 4 sperm (← partially damaged head membrane, deformed and swollen midpiece, damaged flagellum). (**F**) SEM of head and midpiece morphology of Group 6 sperm (← severely damaged head membrane, detached midpiece, broken or detached flagellum).

The ultrastructure of *C. alburnus* sperm was examined by SEM (Figure 4). The spermatozoon is of the typical teleost flagellated type, comprising a spherical, acrosomeless head, a short midpiece, and a long flagellum.

Comparative analysis revealed distinct morphological damage following cryopreservation. Sperm in the G1 displayed intact, smooth morphology. Damage patterns observed in G4 included partial rupture of the plasma membrane over the sperm head, swelling and deformation of the midpiece, and localized flagellar coiling or breakage. Damage patterns in G6 included extensive plasma membrane disintegration in the head region, frequent detachment of the midpiece from the head, and pervasive flagellar damage characterized by severe breakage or complete detachment. Thus, G6 exhibited additional damage features not observed in G4.

#### 3.3.2. TEM Observation of Sperm Ultrastructure

**Figure 5:**
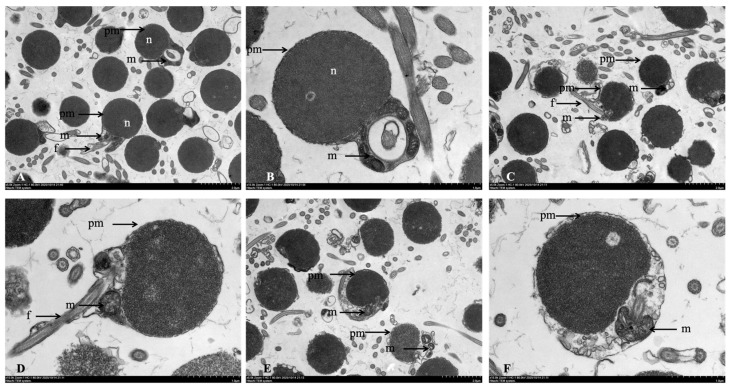
TEM observation of *C. alburnus* sperm ultrastructure. (**A**). TEM of Group 1 sperm (→ normal plasma membrane (pm), mitochondria (m), nucleus (n), and flagellum (f)). (**B**). TEM of Group 1 sperm (→ normal pm, m, and n). (**C**). TEM of Group 4 sperm (→ damaged pm and m, and normal f). (**D**). TEM of Group 4 sperm (→ slightly damaged pm and m, and normal f). (**E**). TEM of Group 6 sperm (→ damaged and deformed pm, lost cytoplasm, damaged m, and the large gap between the m and the pm). (**F**). TEM of Group 6 sperm (→ damaged and deformed pm and m, and the large gap between the m and the pm).

The ultrastructure integrity of sperm was further analyzed by TEM (Figure 5). Sperm from G1 predominantly exhibited a normal architecture. In contrast, a higher incidence of structural damage was observed in the cryopreserved groups (G4 and G6).

In normal sperm (G1), the plasma membrane and nucleus were intact, with condensed chromatin and a discernible perinuclear space. The midpiece contained a cylindrical cytoplasmic canal surrounding the axoneme, populated by mitochondria of varying sizes. Cryopreservation induced distinct ultrastructural alterations. Compared to G1, sperm from both G4 and G6 exhibited plasma membrane rupture or blebbing, cytoplasmic loss, organelle disorganization, and widening of the perinuclear space. Mitochondria were notably swollen, with disrupted cristae. Detachment of the flagellum from the head–midpiece complex was frequently observed.

Differences between G4 and G6 comprised the damaged features observed in G4, which included partial membrane rupture and mitochondrial swelling. In G6, additional damage features were observed, including extensive membrane disintegration and flagellar fracture.

We acknowledge that these TEM observations are based on limited samples with inconsistent sectioning planes and lack quantitative biological replication. Therefore, these findings should be considered preliminary and interpreted as qualitative visual context for the functional and biochemical data, rather than as definitive quantitative evidence of ultrastructural differences.

In conclusion, ultrastructural observation revealed distinct patterns of damage. Sperm in G6 exhibited severe structural lesions including extensive membrane disintegration and flagellar fracture, while damage features in G4 were limited to partial membrane rupture and mitochondrial swelling. These observations provide a qualitative visual correlation with the differences in sperm function and biochemistry observed between groups.

### 3.4. Proteomic Profiling of Sperm

#### 3.4.1. Overview of Protein Identification

Mass spectrometry analysis identified a total of 4760 proteins from 48,844 unique peptides across all sperm samples, providing a comprehensive proteomic profile.

#### 3.4.2. Screening of Differentially Expressed Proteins (DEPs)

**Figure 6:**
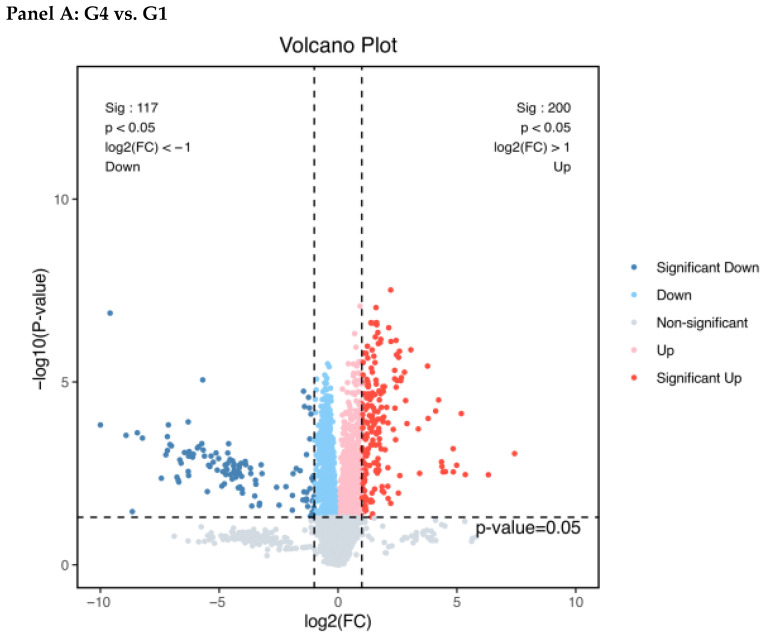

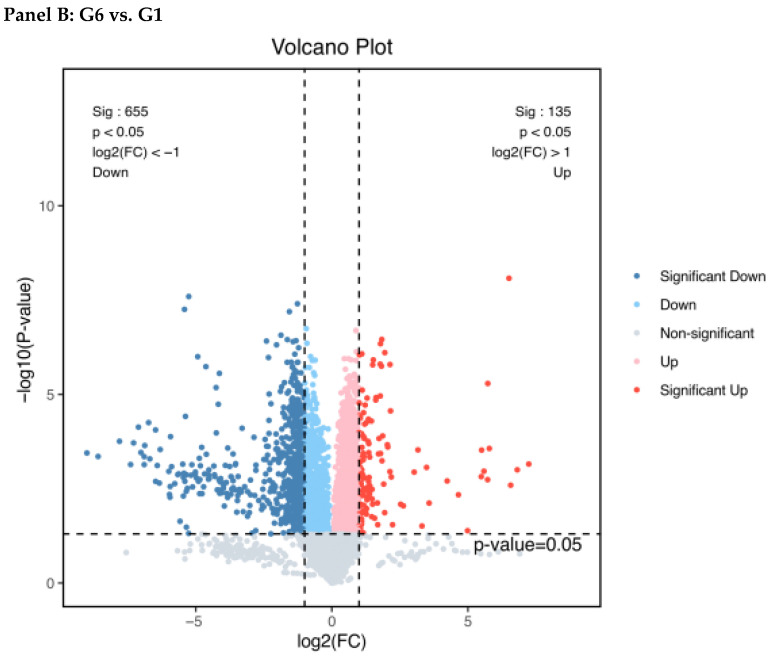
Analysis result of differentially abundant proteins between the proteomes of fresh and cryopreserved sperm of *C. alburnus* (Panel (**A**): number of upregulated and downregulated DEPs in Group 4 (100 mmol/L trehalose) vs. Group 1 (fresh). Panel (**B**): number of upregulated and downregulated DEPs in Group 6 (no cryoprotectant) vs. Group 1). Log2 (sperm expression level of Group 4 or 6/Group 1) was taken as the horizontal axis and −log10 (*p*-value) as the vertical axis. The red dots represent upregulated, significantly differentially expressed proteins. The blue dots represent significantly differentially expressed downregulated proteins, and the further away from point 0, the greater the difference. Gray represents protein with no differential changes.

Using thresholds of |Fold Change| ≥ 2 and a *p*-value < 0.05, we identified DEPs in cryopreserved sperm relative to Group 1. Group 4 (G4, 100 mmol/L trehalose) contained 317 DEPs (200 were present at higher abundance and 117 at lower abundance). In stark contrast, Group 6 (G6, no cryoprotectant) exhibited a much larger proteomic perturbation with 790 DEPs (135 upregulated, 655 downregulated) (Figure 6). This quantitative difference underscores the greater global proteomic disruption caused by cryopreservation in the absence of an effective cryoprotectant.

To dissect the effects of cryodamage and protection, we first compared each cryopreserved group (G4 and G6) to the fresh control (G1). This established the baseline proteomic alterations induced by cryopreservation in both the presence and absence of optimal trehalose protection.

#### 3.4.3. Comparative Functional Enrichment of DEPs

**Figure 7:**
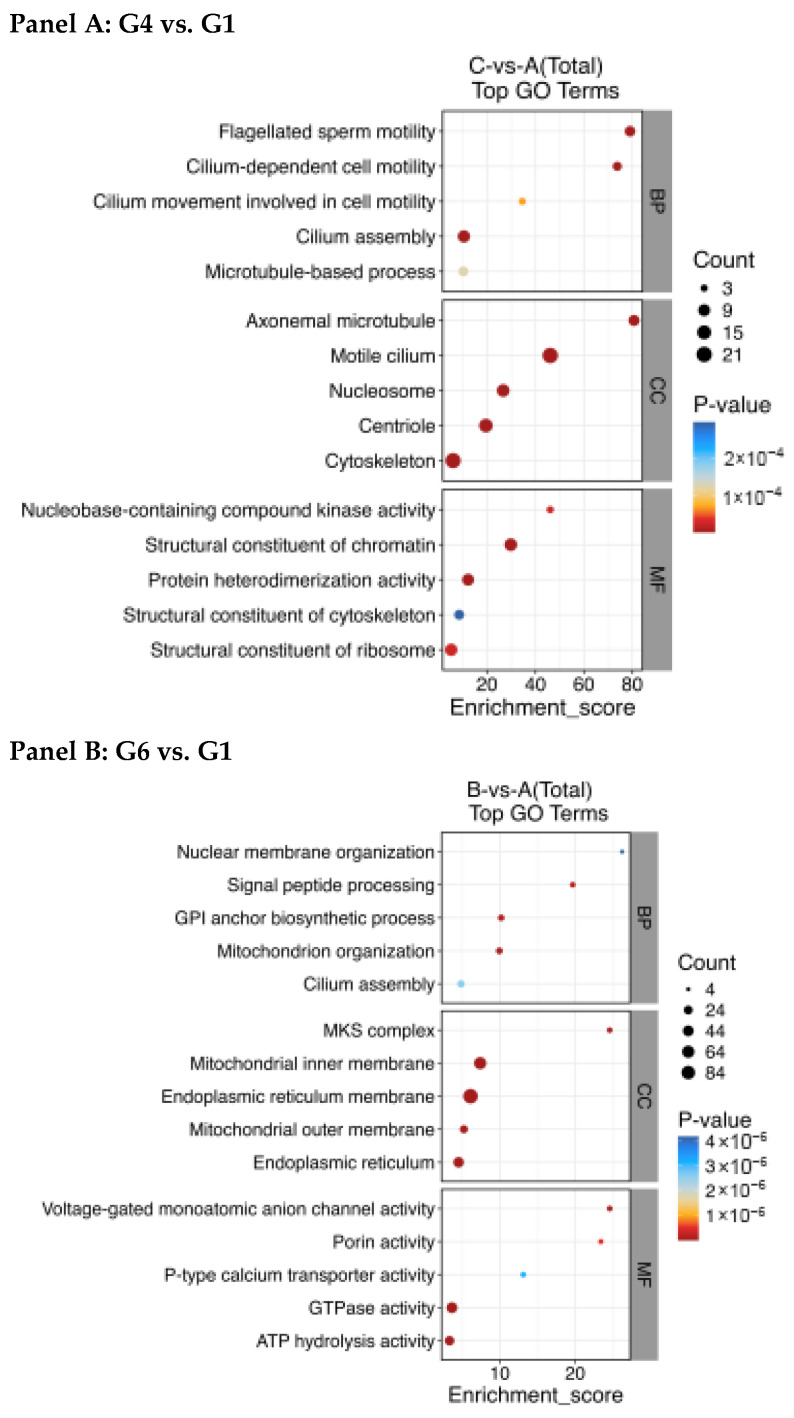
Protein domain analysis of differentially abundant proteins (Panel (**A**): Top 15 enriched GO terms in Group 4 (100 mmol/L trehalose) vs. Group 1 (fresh). Panel (**B**): Top 15 enriched GO terms in Group 6 (no cryoprotectant) vs. Group 1). The red, blue and grey dots represent significantly upregulated, downregulated and not significantly changed proteins, respectively. The further away the dot is from the point 0, the greater the difference.

**Figure 8:**
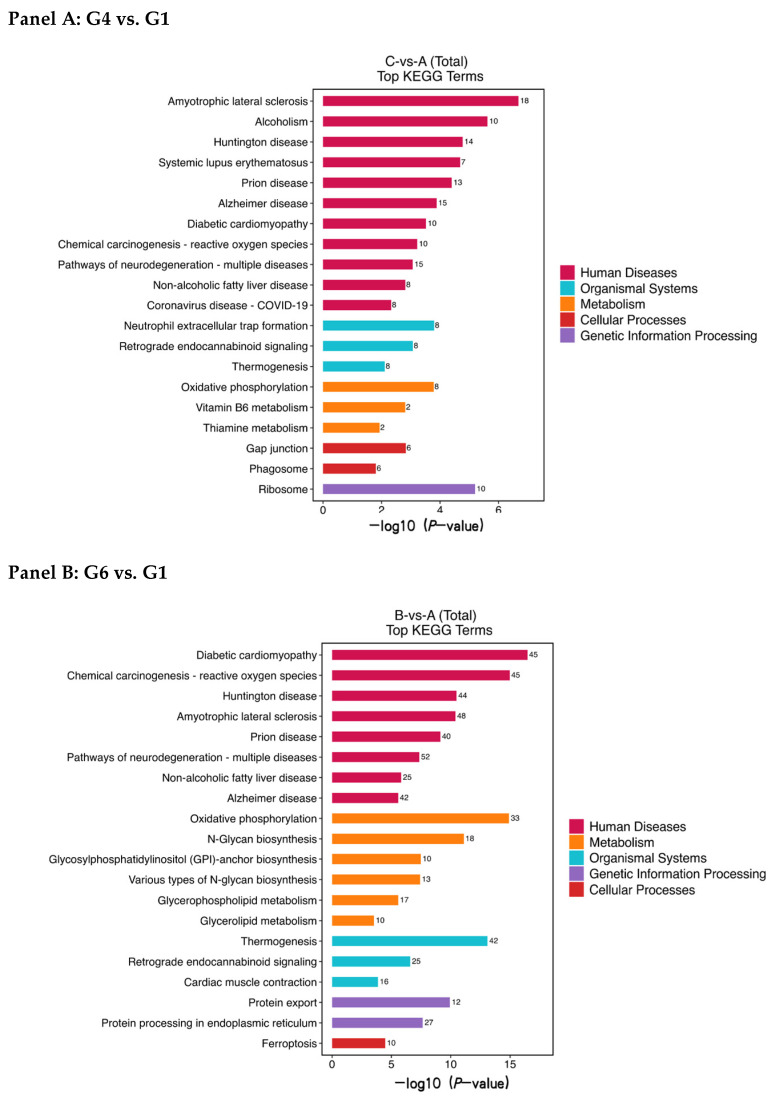
The distribution of KEGG pathways of differential proteins (Panel (**A**): Top enriched pathways in Group 4 (100 mmol/L trehalose) vs. Group 1 (fresh). Panel (**B**): Top enriched pathways in Group 6 (no cryoprotectant) vs. Group 1).

To elucidate the distinct biological states associated with protection (G4) and severe damage (G6), we performed GO and KEGG enrichment analyses on their respective DEPs. The results revealed fundamentally different functional themes (Figure 7 and Figure 8, Table 1 and Table 2).

Compared to fresh sperm (Group 1), the DEPs in Group 4 (100 mmol/L trehalose, protected state) were significantly enriched in terms related to cellular structure and motility. Key enriched CC (cellular components) included the motile cilium, axoneme, cytoskeleton, and nucleosome. Corresponding BP (biological processes) involved sperm motility, cilium assembly, and microtubule-based processes. MF (molecular functions) were related to chromatin and cytoskeletal structural constituents. This pattern suggests that the proteomic response in G4 is primarily geared towards preserving the structural integrity of the motility apparatus and chromatin organization.

In contrast, DEPs in Group 6 (no cryoprotectant, damaged state) were overwhelmingly enriched in terms associated with cellular stress, organelle damage, and metabolic dysfunction. Enriched CC prominently featured the endoplasmic reticulum (ER) membrane, mitochondrial membranes, and the MKS complex. MF included GTPase activity, ATP hydrolysis activity, and ion transporter activity. BP centered on mitochondrion organization, ER-related processes (e.g., signal peptide processing), and cilium assembly—the latter likely reflecting disrupted biogenesis. This profile indicates widespread organelle (ER and mitochondria) stress, disrupted energy metabolism, and compromised ion homeostasis.

#### 3.4.4. Key Signaling Pathways and Candidate Proteins

KEGG pathway analysis further highlighted these divergent states (Figure 8, Table 3 and Table 4). In G4 vs. G1, notable pathways included amyotrophic lateral sclerosis, alcoholism, ribosome, etc. In G6 vs. G1, pathways such as diabetic cardiomyopathy, chemical carcinogenesis-ROS, oxidative phosphorylation, and thermogenesis were highly enriched, reinforcing the themes of metabolic stress and proteotoxicity.

From the most significantly enriched pathways, we identified several key candidate proteins whose abundance patterns differed between groups:

In G4 vs. G1: *Histone H2A* (chromatin stability), *cytochrome c oxidase (COX)* and *ubiquinol-cytochrome C reductase (UQCRB)* (mitochondrial electron transport), and *profilin* (cytoskeletal dynamics) were present at higher relative abundance.

In G6 vs. G1: *NADH dehydrogenase* (mitochondrial complex I) was present at lower abundance, indicating impaired electron transport chain initiation. Conversely, *calcium-transporting ATPase (PMCA)* and *glutathione S-transferase (GST)* were present at higher abundance, likely representing compensatory responses to calcium dysregulation and elevated oxidative stress, respectively.

The contrasting abundance patterns of these proteins between G4 and G6 provide molecular insights into the differences between the two cryopreservation conditions. However, we note that without direct statistical comparisons between G4 and G6 (or G4 vs. G2), these patterns should be interpreted as candidate associations rather than definitive mechanistic evidence.

It is important to note that GO and KEGG enrichment analyses annotate proteins based on functional information derived primarily from somatic cell studies. In the context of mature spermatozoa, which are transcriptionally and translationally inactive, these annotations should be interpreted as indicators of the proteins’ known molecular functions, rather than as evidence of active biological processes occurring during cryopreservation. For example, terms such as ‘translation’ and ‘nucleosome assembly’ reflect the presence of ribosomal or chromatin-associated proteins (e.g., *Histone H2A*), not ongoing translational activity or nucleosome remodeling.

## 4. Discussion

### 4.1. The Impact of Cryopreservation on Sperm Enzyme Activity and the Protective Role of Trehalose

The present study demonstrates that cryopreservation induces significant alterations in the enzymatic profile of *C. alburnus* sperm. A pronounced decline was observed in both antioxidant enzymes (SOD, CAT) and key enzymes involved in energy metabolism (ATPase, SDH, LDH). The formulation containing 100 mmol/L trehalose (Group 4) was associated with attenuated decline in these activities compared to other cryopreserved groups, preserving them at levels closest to those of fresh sperm. This protective effect aligns with previous findings on trehalose-supplemented cryomedium in other fish models [11].

The decrease in antioxidant enzyme activities (SOD, CAT) post-thaw indicates a compromised cellular defense against oxidative stress, a well-established hallmark of cryodamage [30,31]. During freezing, the overproduction of ROS coupled with impaired antioxidant systems can lead to lipid peroxidation, protein oxidation, and DNA fragmentation, ultimately diminishing sperm viability [32]. Our results confirm that cryopreservation overwhelms the sperm’s intrinsic antioxidant capacity. The higher SOD and CAT activities observed in G4 suggest that the trehalose-supplemented formulation contributes to preserving redox balance, potentially through membrane stabilization that reduces enzyme leakage or by protecting enzyme structure itself. However, it should be noted that fertilization in fish typically occurs rapidly (often within seconds of gamete contact). Under these conditions, the biological relevance of long-term oxidative stress protection may be limited. Therefore, the observed differences in antioxidant enzyme activities should be interpreted primarily as indicators of general cellular integrity during cryopreservation rather than as direct predictors of fertilization success.

Similarly, the depression of energy metabolism enzymes reflects a profound disturbance in bioenergetic homeostasis. The observed reductions in ATPase, SDH, and LDH activities are consistent with reports in species such as *Monopterus albus* [16], *Anguilla japonica* [33], and *Acrossocheilus fasciatus* [34]. These enzymes are integral to ATP synthesis, mitochondrial electron transport, and glycolysis, respectively. Their diminished activity correlates with reduced capacity for ATP production, which is essential for flagellar movement and post-thaw motility [35]. The higher residual activities of these enzymes in G4 are consistent with the mitochondrial swelling patterns observed in this group (Section 3.3).

It is important to note that enzyme activities measured in sperm pellets may be influenced by leakage through damaged membranes, as cryopreservation-induced structural damage can lead to loss of intracellular enzymes. Therefore, lower activities in damaged sperm (G6) may reflect both functional impairment and enzyme loss, while higher activities in protected sperm (G4) may indicate both better-preserved intracellular function and reduced leakage. The observed differences should thus be interpreted as integrative indicators of overall sperm integrity rather than strictly intracellular functional status.

The underlying mechanism for the loss of enzymatic activity likely involves combined physical and biochemical insults. Ice crystals formation and dissolution can cause mechanical disruption of cellular and organellar membranes, potentially leading to the leakage of enzymes, as proposed in studies on other species [16,36]. Additionally, cryopreservation-induced denaturation or conformational changes in the enzymes themselves might contribute to activity loss. Our data suggest that trehalose, through its water-replacement and vitrifying properties [9,10], may stabilize membrane structures and denaturation of critical enzymes.

In conclusion, the enzymatic assays indicate that 100 mmol/L trehalose supplemented formulation is associated with better preservation of antioxidant defenses and energy metabolism potential in post-thaw *C. alburnus* sperm. These findings correlate with the functional benefit observed in motility assays and identify oxidative stress response and energy metabolism as pathways potentially involved in improved cryopreservation outcomes. Future strategies to enhance cryopreservation protocols could involve the synergistic use of trehalose with specific antioxidants (e.g., SOD, CAT) to further fortify these vulnerable systems, as preliminary success has been shown in ram and trout sperm [37,38]. In addition, membrane integrity assays (e.g., SYBR-14/PI staining) and fractionated enzyme measurements (supernatant vs. pellet) would help distinguish between functional impairment and leakage artifacts, providing a more precise mechanistic understanding.

### 4.2. Ultrastructural Alterations Induced by Cryopreservation

Our ultrastructural analysis via SEM and TEM provides direct visual evidence of the physical damage inflicted on *C. alburnus* sperm by the freeze–thaw cycle. The observed spectrum of injury ranging from plasma membrane rupture and midpiece deformation to flagellar coiling or detachment aligns with established hallmarks of cryodamage across fish species [11,20,36]. Notably, damage features observed in G4 included partial membrane rupture and mitochondrial swelling, while G6 exhibited additional damage features including extensive membrane disintegration and flagellar fracture.

The integrity of the sperm head plasma membrane is paramount. Its compromise, as frequently observed in G6, can lead to leakage of intracellular components and render the nucleus vulnerable to osmotic and oxidative stress, potentially affecting genetic integrity and fertilization potential [36]. The midpiece, housing the mitochondria, is the cellular powerhouse. Damage to this compartment, including mitochondrial swelling and cristae disruption, may impair ATP synthesis, affecting the energy supply required for motility [39]. Our observations of less severe mitochondrial alteration in G4 correlate with the preserved SDH activity and higher ATPase levels discussed earlier (Section 3.2), linking structural appearance to functional bioenergetics. Finally, the flagellum is the executioner of movement. Damage, curling, or detachment of the flagellum would physically compromise sperm motility, as flagellar movement relies on the coordinated sliding of axonemal microtubules powered by mitochondrial ATP [40].

The primary driver of this structural damage is widely attributed to the combined effects of intracellular ice crystal formation and osmotic stress during freezing and thawing [38]. Ice crystals can cause direct mechanical shearing of membranes and organelles. Concurrently, the formation of extracellular ice increases solute concentration, creating osmotic gradients that may cause cell shrinkage and re-expansion, leading to membrane blebbing, rupture, and protein denaturation [41]. Even with cryoprotectants, some degree of membrane perturbation is often unavoidable, as noted in studies on *A. schrenckii* [11] and *M. amblycephala* [18], which report membrane wrinkling, vesiculation, and loss of surface proteins.

The apparent structural preservation in G4 may be related to the biophysical properties of trehalose. Its well characterized “water replacement” hypothesis posits that trehalose molecules hydrogen bond to phospholipid head groups and membrane proteins, substituting for water molecules lost during dehydration and preventing the phase transition and fusion of membranes [9,10]. This action may help stabilize the plasma membrane, mitochondrial membranes, and the flagellar sheath. Additionally, by promoting vitrification in the extracellular matrix, trehalose may suppress ice crystal growth, thereby reducing mechanical injury.

We fully acknowledge that our ultrastructural observations are qualitative and based on representative images rather than quantitative assessment across a statistically meaningful number of sperm. Although extensive microscopic examination confirmed that the presented images reflect the predominant morphology observed within each group, this approach does not constitute rigorous quantitative analysis and cannot definitively exclude potential artifacts or selection bias. Therefore, these observations should be interpreted as visual context for the functional and biochemical data rather than as definitive structural evidence.

In conclusion, the ultrastructural observations provide visual evidence that aligns with the functional and biochemical differences among treatment groups. Sperm cryopreserved with the trehalose-supplemented formulation (G4) exhibited damage features limited to partial plasma membrane rupture and mitochondria swelling, consistent with their higher motility parameters and better-preserved enzyme activities. These findings suggest that structural preservation may contribute to maintaining post-thaw sperm function, although fertilization assays are required to confirm this relationship. The observed correlation between structural integrity and functional outcomes supports the potential utility of trehalose as a protective component in cryopreservation protocols for this species. Future studies should incorporate quantitative morphometric analysis, examining a statistically robust number of sperm per group (e.g., *n* > 100) with blinded assessment and categorization of specific abnormalities, followed by appropriate statistical comparisons.

### 4.3. Proteomic Insights into Cryodamage and Cryoprotection

Having discussed the functional and structural findings, we now turn to the proteomic data, which offer molecular-level insights. Proteomic analysis revealed profound, yet distinctly different, alterations in the sperm proteome induced by cryopreservation. The scale of disruption was quantitatively greater in the severely damaged Group 6 (790 DEPs) compared to the protected Group 4 (317 DEPs), consistent with the graded severity observed in functional and structural assays.

#### 4.3.1. Molecular Signature of Trehalose-Associated Protection (Group 4)

The proteomic profile of Group 4 reflects differential preservation of proteins involved in cellular integrity. The higher relative abundance of *Histone H2A* suggests that this protein is better protected from degradation or leakage during cryopreservation, potentially contributing to chromatin stability [42]. Concurrently, the elevated abundance of mitochondrial electron transport chain components (*COX* and *UQCRB*) indicates maintained capacity for oxidative phosphorylation, which is consistent with the higher SDH and ATPase activities observed in this group [43,44,45]. Furthermore, the higher abundance of profilin, a regulator of actin and potentially tubulin dynamics, alongside STPPs involved in motility regulation, highlights preservation of the cytoskeletal architecture essential for flagellar function [44,46].

#### 4.3.2. Molecular Hallmarks of Severe Cryodamage (Group 6)

In stark contrast, the proteome of Group 6 depicts a state of metabolic crisis and severe oxidative stress. The lower relative abundance of *NADH dehydrogenase* indicates that this protein is particularly susceptible to degradation or loss during cryopreservation in the absence of adequate protection, contributing to impaired mitochondrial electron transport [47]. This is accompanied by the higher abundance of *PMCA*, potentially reflecting a compensatory response to calcium homeostasis disruption, and *GST*, a marker of elevated oxidative stress [48,49]. The altered abundance of *small monomeric GTPases*, key signaling molecules, further suggests widespread disruption of cellular signaling and survival pathways [50].

#### 4.3.3. Integration with Functional Data and Interpretational Caveats

The proteomic findings are coherently linked with other layers of data. The lower abundance of motility related proteins and GTPases in Group 6 correlates with its poor post-thaw motility and severe flagellar defects observed by SEM/TEM, a phenomenon also reported in *A. schrenckii* [32]. Conversely, the higher abundance of structural and metabolic proteins in Group 4 aligns with its superior motility, preserved enzyme activities, and ultrastructure.

Several interpretational caveats must be emphasized. First, mature spermatozoa are transcriptionally and translationally inactive. Therefore, the observed differences in protein abundance reflect differential protein stability, degradation, or leakage during freeze–thaw, rather than active gene expression changes. Second, our proteomic analysis compared each cryopreserved group separately to fresh sperm (G1). While this approach identifies proteins altered by cryopreservation under different conditions, it does not allow us to distinguish between general cryopreservation-induced changes and those specifically associated with trehalose supplementation. Direct comparisons between G4 vs. G6 and G4 vs. G2 would be required to isolate protection-specific changes from background cryopreservation effects. Third, our experimental design, comparing the G4 to G6, cannot separate the individual contributions of trehalose from those of EG. The improved outcomes observed in G4 should therefore be attributed to the combined action of both cryoprotectants.

#### 4.3.4. Candidate Protein Biomarkers

Despite these limitations, the proteomic analysis identifies candidate protein biomarkers that distinguish cryopreservation outcomes: *NADH dehydrogenase, PMCA,* and *GST* as signatures of severe damage; *UQCRB* and *profilin* as indicators of successful protection. These proteins, particularly those governing mitochondrial function, redox balance, and cytoskeletal integrity, represent potential targets for assessing cryopreservation success. The enrichment of KEGG pathways labeled with human disease terms (e.g., Alzheimer disease) reflects the presence of conserved homologous proteins that participate in fundamental cellular functions such as oxidative phosphorylation, proteostasis, and apoptosis in other cell types. In the context of mature spermatozoa, these annotations should not be interpreted as evidence of disease-related or active pathway engagement; rather, they indicate that proteins with these annotated functions were differentially abundant between groups. Similarly, terms such as “translation” and “nucleosome assembly” reflect the presence of ribosomal proteins and histones (e.g., *Histone H2A*), which may play structural or protective roles during cryopreservation, rather than indicating active protein synthesis or chromatin remodeling.

#### 4.3.5. Limitations and Future Directions

Several methodological limitations should be acknowledged. First, the pathway-level associations reported here are correlative rather than causal; identification of proteins associated with protection does not prove functional necessity. Second, the absence of a direct proteomic comparison (G4 vs. G6 and G4 vs. G2) limits our ability to attribute specific protein changes to trehalose itself versus general cryopreservation effects. Third, our experimental design lacks an EG-only control group, preventing definitive separation of trehalose and EG contributions. Finally, the candidate biomarkers identified here require validation against actual fertilization outcomes to establish their predictive value. Future studies should address these limitations through direct proteomic comparison to isolate protection-specific changes, include EG-only controls, target the validation of candidate proteins (e.g., PRM, Western blot), employ CASA for more objective motility assessment, and control fertilization trials. Such efforts will be essential to move from correlation to causation and to develop targeted strategies for improving cryopreservation protocols.

### 4.4. Implications for Sperm Cryopreservation in C. alburnus

Building upon previous work from our team that established a baseline cryomedium (D-15 with 10% EG) for *C. alburnus* sperm [4,5], the present study aimed to further enhance post-thaw quality by supplementing this base formulation with trehalose. Trehalose was selected based on its renowned cryoprotective properties, which stem from its unique ability to stabilize phospholipid bilayers and proteins during dehydration and freezing, primarily through the “water replacement” mechanism [9,51]. Our results indicate that the formulation supplementing with 100 mmol/L trehalose was associated with improved outcomes across motility, biochemistry, ultrastructure, and proteomic profiles compared to the unprotected control (G6). However, we note that fertilization trials are necessary to determine whether these improvements translate to enhanced reproductive success.

The concentration of 100 mmol/L identified here situates well within the effective range reported across diverse taxa. It is consistent with concentrations used in mammalian models (e.g., 50–100 mmol/L for goat and cattle sperm [52,53]) and aligns with studies on fish such as *A. schrenckii* and *Epinephelus* spp. (60–90 mmol/L) [10,11,54], while being lower than the higher concentrations required for certain crustacean sperm [55,56]. These comparisons suggest that trehalose efficacy is both species- and cell type-dependent. However, we note that because our experimental design lacked an EG-only control group, the improved outcomes observed in G4 reflect the combined action of EG and trehalose, and the individual contribution of trehalose cannot be isolated from this study.

Beyond identifying a candidate formulation, this study underscores the importance of a multi-parametric analytical framework for evaluating cryopreservation success. Motility analysis provides the ultimate functional readout but lacks mechanistic insight. By integrating enzyme activity assays, we obtained evidence linking the trehalose-supplemented formulation to preservation of antioxidant defenses (SOD, CAT) and energy metabolism (ATPase, SDH, LDH). Ultrastructural visualization (SEM/TEM) offered unambiguous proof of its membrane- and organelle-stabilizing effects, correlating structural preservation with functional resilience. Finally, proteomic profiling delivered a systems-level understanding, revealing distinct molecular signatures associated with the trehalose-supplemented formulation (e.g., higher abundance of *UQCRB*, *profilin*) versus severe damage (e.g., lower abundance of *NADH dehydrogenase*). The convergence of these datasets strengthens overall interpretation and provides a holistic view of cryodamage pathways.

Therefore, we suggest that the concurrent application of these techniques offers a useful framework for developing and refining cryopreservation protocols. Enzyme activity and ultrastructural analysis can serve as intermediate indicators of sperm quality. Proteomics, in turn, can identify candidate molecular targets and predictive biomarkers, such as those highlighted in this study, enabling more rational design of cryoprotectant formulations.

In conclusion, this research advances the germplasm preservation technology for *C. alburnus* by identifying a candidate formulation containing 100 mmol/L trehalose that was associated with improved cryopreservation outcomes. More broadly, it demonstrates a powerful integrative methodology that deciphers the mechanisms of cryoprotection. Future studies should employ this multi-omics and multi-scale framework to further explore synergistic additives, refine freezing protocols, and ultimately ensure the reliable long term conservation of genetic resources for sustainable aquaculture.

## 5. Conclusions

This study establishes that supplementing a conventional cryomedium (D-15 + 10% EG) with 100 mmol/L trehalose was associated with improved post-thaw quality of *C. alburnus* sperm. This formulation might enhance motility parameters, better-preserved enzyme activities, and reduced ultrastructural damage to the plasma membrane, mitochondria, and flagella.

Proteomic analysis revealed that this improved outcome was associated with higher relative abundance of proteins involved in chromatin stability (*Histone H2A*), mitochondrial function (*COX*, *UQCRB*), and cytoskeletal dynamics (*profilin*, *STPPs*), contrasting with lower abundance of core metabolic proteins (*NADH dehydrogenase*) and higher abundance of stress marker (*PMCA*, *GST*) in unprotected sperm. These proteins represent candidate biomarkers for assessing cryopreservation outcomes.

We acknowledge that validation through direct comparison with EG-only controls and fertilization trials is required before this protocol can be considered for aquaculture applications. Additionally, the proteomic findings are correlational and should be integrated with caution given the transcriptional inactivity of mature sperm. Despite these limitations, this integrated multi-parametric approach, combining motility assays, enzyme activity tests, ultrastructural examination, and proteomic profiling, provides a framework for further refinement of germplasm cryopreservation technologies in this species.

## Figures and Tables

**Table 1:** GO function enrichment analysis of differential proteins in Group 4 (100 mmol/L trehalose) vs. Group 1 (fresh sperm). Top 30 enriched terms are shown for cellular component, molecular function, and biological process.

Group 4 vs. 1 GO No.	Group 4 vs. 1 Pathway Name	Group 4 vs. 1 Category
GO:0031514	Motile cilium	Cellular component
GO:0005814	Centriole	Cellular component
GO:0030527	Structural constituent of chromatin	Molecular function
GO:0005879	Axonemal microtubule	Cellular component
GO:0000786	Nucleosome	Cellular component
GO:0030317	Flagellated sperm motility	Biological process
GO:0005856	Cytoskeleton	Cellular component
GO:0060271	Cilium assembly	Biological process
GO:0046982	Protein heterodimerization activity	Molecular function
GO:0036126	Sperm flagellum	Cellular component
GO:0060285	Cilium-dependent cell motility	Biological process
GO:0005929	Cilium	Cellular component
GO:0005930	Axoneme	Cellular component
GO:0036064	Ciliary basal body	Cellular component
GO:0003735	Structural constituent of ribosome	Molecular function
GO:0019205	Nucleobase-containing compound kinase activity	Molecular function
GO:0060294	Cilium movement involved in cell motility	Biological process
GO:0007017	Microtubule-based process	Biological process
GO:0006412	Translation	Biological process
GO:0015630	Microtubule cytoskeleton	Cellular component
GO:0044782	Cilium organization	Biological process
GO:0005743	Mitochondrial inner membrane	Cellular component
GO:0005200	Structural constituent of cytoskeleton	Molecular function
GO:0003352	Regulation of cilium movement	Biological process
GO:0019104	DNA N-glycosylase activity	Molecular function
GO:0060287	Epithelial cilium movement involved in determination of left/right asymmetry	Biological process
GO:0006334	Nucleosome assembly	Biological process
GO:0006139	Nucleobase-containing compound metabolic process	Biological process
GO:0035082	Axoneme assembly	Biological process

**Table 2:** GO function enrichment analysis of differential proteins in Group 6 (no cryoprotectant) vs. Group 1 (fresh sperm). Top 30 enriched terms are shown for cellular component, molecular function, and biological process.

Group 6 vs. 1 GO No.	Group 6 vs. 1 Pathway Name	Group 6 vs. 1 Category
GO:0005789	Endoplasmic reticulum membrane	Cellular component
GO:0005743	Mitochondrial inner membrane	Cellular component
GO:0005783	Endoplasmic reticulum	Cellular component
GO:0003924	GTPase activity	Molecular function
GO:0005741	Mitochondrial outer membrane	Cellular component
GO:0016887	ATP hydrolysis activity	Molecular function
GO:0008308	Voltage-gated monoatomic anion channel activity	Molecular function
GO:0036038	MKS complex	Cellular component
GO:0007005	Mitochondrion organization	Biological process
GO:0031514	Motile cilium	Cellular component
GO:0006506	GPI anchor biosynthetic process	Biological process
GO:0006465	Signal peptide processing	Biological process
GO:0015288	Porin activity	Molecular function
GO:0008250	Oligosaccharyltransferase complex	Cellular component
GO:0031966	Mitochondrial membrane	Cellular component
GO:0005787	Signal peptidase complex	Cellular component
GO:0016600	Flotillin complex	Cellular component
GO:0060271	Cilium assembly	Biological process
GO:0046930	Pore complex	Cellular component
GO:0005388	P-type calcium transporter activity	Molecular function
GO:0071763	Nuclear membrane organization	Biological process
GO:0005637	Nuclear inner membrane	Cellular component
GO:0005525	GTP binding	Molecular function
GO:0018279	Protein N-linked glycosylation via asparagine	Biological process
GO:0035101	FACT complex	Cellular component
GO:0005635	Nuclear envelope	Cellular component
GO:0006487	Protein N-linked glycosylation	Biological process
GO:0016020	Membrane	Cellular component
GO:0006120	Mitochondrial electron transport, NADH to ubiquinone	Biological process
GO:0055085	Transmembrane transport	Biological process

**Table 3:** KEGG pathway enrichment analysis of differentially expressed proteins in Group 4 (100 mmol/L trehalose) vs. Group 1 (fresh sperm). Top 10 enriched pathways and associated candidate proteins are shown.

Group 4 vs. 1 KEGG Pathway	Group 4 vs. 1 Protein
Amyotrophic lateral sclerosis	Cytochrome c oxidase, profilin, ubiquinol-cytochrome C reductase hinge domain-containing protein
Alcoholism	Histone H2A, guanine nucleotide-binding protein, serine/threonine-protein phosphatase
Ribosome	Mitochondrial ribosomal protein S30, 60S ribosomal protein L36a
Huntington disease	Cytochrome c oxidase, TATA-box-binding protein, tubulin beta chain
Systemic lupus erythematosus	Histone H2A, core histone macro-H2A, histone H4
Prion disease	Cytochrome c oxidase, tubulin beta chain, NADH dehydrogenase
Alzheimer disease	Cytochrome c oxidase, tubulin beta chain, tubulin alpha chain
Neutrophil extracellular trap formation	Histone H2A, histone H4, histone H2B
Oxidative phosphorylation	Cytochrome c oxidase, NADH dehydrogenase, ubiquinol-cytochrome C reductase hinge domain-containing protein
Diabetic cardiomyopathy	Cytochrome c oxidase, serine/threonine-protein phosphatase, NADH dehydrogenase

**Table 4:** KEGG pathway enrichment analysis of differentially expressed proteins in Group 6 (no cryoprotectant) vs. Group 1 (fresh sperm). Top 10 enriched pathways and associated candidate proteins are shown.

Group 6 vs. 1 KEGG Pathway	Group 6 vs. 1 Protein
Diabetic cardiomyopathy	NADH dehydrogenase, calcium-transporting ATPase, cytochrome c oxidase
Chemical carcinogenesis—reactive oxygen species	Small monomeric GTPase, NADH dehydrogenase, glutathione S-transferase, mitochondrial
Oxidative phosphorylation	NADH dehydrogenase, cytochrome c oxidase, V-type proton ATPase subunit
Thermogenesis	Small monomeric GTPase, NADH dehydrogenase, long-chain-fatty-acid-CoA ligase
N-Glycan biosynthesis	Dolichol-phosphate mannosyltransferase, dolichyl-diphosphooligosaccharide-protein glycosyltransferase
Huntington disease	NADH dehydrogenase, dynein axonemal heavy chain, ATPase domain-containing protein
Amyotrophic lateral sclerosis	NADH dehydrogenase, dynein axonemal heavy chain, cytochrome c oxidase
Protein export	Protein transport protein Sec61, translocation protein SEC63 homolog, translocation protein SEC62
Prion disease	NADH dehydrogenase, complement component C6, cytochrome c oxidase
Protein processing in endoplasmic reticulum	Protein transport protein Sec61, translocating chain-associated membrane protein, small monomeric GTPase

## Data Availability

The data and materials are available on request.
